# Human Cytomegalovirus Gene UL76 Induces IL-8 Expression through Activation of the DNA Damage Response

**DOI:** 10.1371/journal.ppat.1003609

**Published:** 2013-09-12

**Authors:** Helena Costa, Rute Nascimento, John Sinclair, Robert Michael Evans Parkhouse

**Affiliations:** 1 Instituto Gulbenkian de Ciência, Oeiras, Portugal; 2 Department of Medicine, University of Cambridge, Cambridge, United Kingdom; University of Alabama at Birmingham, United States of America

## Abstract

Human cytomegalovirus (HCMV), a β-herpesvirus, has evolved many strategies to subvert both innate and adaptive host immunity in order to ensure its survival and propagation within the host. Induction of IL-8 is particularly important during HCMV infection as neutrophils, primarily attracted by IL-8, play a key role in virus dissemination. Moreover, IL-8 has a positive effect in the replication of HCMV. This work has identified an HCMV gene (UL76), with the relevant property of inducing IL-8 expression at both transcriptional and protein levels. Up-regulation of IL-8 by UL76 results from activation of the NF-kB pathway as inhibition of both IKK-β activity or degradation of Ikβα abolishes the IL-8 induction and, concomitantly, expression of UL76 is associated with the translocation of p65 to the nucleus where it binds to the IL-8 promoter. Furthermore, the UL76-mediated induction of IL-8 requires ATM and is correlated with the phosphorylation of NEMO on serine 85, indicating that UL76 activates NF-kB pathway by the DNA Damage response, similar to the impact of genotoxic drugs. More importantly, a UL76 deletion mutant virus was significantly less efficient in stimulating IL-8 production than the wild type virus. In addition, there was a significant reduction of IL-8 secretion when *ATM -/-* cells were infected with wild type HCMV, thus, indicating that ATM is also involved in the induction of IL-8 by HCMV.

In conclusion, we demonstrate that expression of UL76 gene induces IL-8 expression as a result of the DNA damage response and that both UL76 and ATM have a role in the mechanism of IL-8 induction during HCMV infection. Hence, this work characterizes a new role of the activation of DNA Damage response in the context of host-pathogen interactions.

## Author Summary

The importance of herpesviruses is evident by their prevalence in the human population and the diverse range of diseases that they provoke. Their ability to establish latency provides a compelling example of how herpesviruses successfully evade the immune system and manipulate cellular biology. One promising approach for the development of novel anti-viral strategies is to study viral proteins involved in these host-pathogen interactions. One example is the induction of the pro-inflammatory chemokine IL-8 by HCMV which enhances viral replication and dissemination of the virus by neutrophils. Here, we have identified HCMV UL76 gene, conserved in all herpesviruses, as an inducer of IL-8, and thus with an important impact on HCMV pathogenesis. The induction of IL-8 by UL76 results from activation of the DNA Damage response, which may also explain why UL76 also induces cell cycle arrest. These findings are a clear example of how viruses manipulate intracellular signaling pathways with different outcomes that will be beneficial for viral infection. Finally, the fact that UL76 is a non-homologous gene substantiates the premise that many such pathogen genes without homology may indeed have evolved for host manipulation, and are a repository of potential useful tools for experimental manipulation in health and disease.

## Introduction

Human cytomegalovirus (HCMV) is a β-herpesvirus that infects healthy individuals, usually asymptomatically, but can cause severe or fatal disease in immunocompromised individuals. Primary HCMV infection, as with other herpesviruses, is followed by establishment of lifelong latency with periodic reactivation and, in order to successfully establish itself in the host, the virus has evolved a broad range of host evasion strategies, modulating not only innate and adaptive immunity, but also host cell biology, for example, the cell cycle and apoptosis [1].

The induction of the interleukin-8 (IL-8) during HCMV infection is particularly important for viral replication and possibly contributes to the efficient dissemination of the virus by neutrophils [2], [3]. Interleukin-8 is a pro-inflammatory chemokine that attracts primarily neutrophils, and also monocytes and cytotoxic T cells, by interacting with the CXC chemokine receptors CXCR1 and CXCR2 [4]. Although expression of IL-8 is low or absent under normal conditions, it is highly inducible by a wide range of extracellular stimuli, such as the pro-inflammatory cytokine IL-1, the tumor necrosis factor alpha (TNFα) [5], bacteria and viruses [6], [7]. Besides its relevant role in inflammation, IL-8 is a key component in several viral infections, modulating viral dissemination and virus replication, in part due to inhibition of the impact of interferon-α [8]. On the other hand, excessive amounts of locally produced IL-8 can have deleterious effects, and so IL-8 gene expression is tightly controlled at both transcriptional and post-transcriptional levels. Activation of IL-8 expression in the majority of cell types is critically controlled by the NF-kB transcription factor. The AP-1 and NF-IL-6 transcription factors may also contribute to optimal IL-8 activation, depending on the stimulus or the cell type [4].

The NF-kB canonical pathway involves the activation of the IKK complex, consisting of two catalytic kinase subunits, IKKα and IKKβ, and a regulatory subunit, IKKγ/NEMO. In most unstimulated cells, NF-kB dimers (mostly p65/p50 dimers) are localized in the cytoplasm as a complex with the IkB proteins. Upon stimulation, IkB is phosphorylated by the IKK complex, ubiquitinated and targeted for degradation, thus releasing the NF-kB subunits that translocate to the nucleus and induce transcription of target genes [9]. Although most of the physiological inducers of NF-kB involve the canonical pathway, alternative mechanisms leading to NF-kB nuclear localization and DNA binding have been identified. One of these pathways is induced by activation of the DNA damage response and, in contrast to inflammatory stimuli such as TNFα or IL-1β, the signal originates in the nucleus [10]. Activation of NF-kB by genotoxic stress requires induction of two independent parallel pathways. The first one triggered upon DNA damage results in the phosphorylation and activation of ATM, a nuclear protein kinase which regulates cell cycle checkpoints in response to DNA double-strand breaks [11]. The second pathway leads to SUMOylation of NEMO through a mechanism dependent on PARP1, PIASy and Ubc9. Activation of both these pathways leads to phosphorylation and ubiquitination of sumoylated NEMO in an ATM-dependent way. Ubiquitinated NEMO associated with ATM is exported back to the cytoplasm, activating the IKK complex and subsequent NF-kB activation in a similar manner to the canonical pathway [10].

The HCMV UL76 protein is virion-associated and expressed with late kinetics [12]. The corresponding gene belongs to the UL24 gene family, conserved in all herpesviruses and the only core gene without an assigned function [13]. Bioinformatic analysis identified UL24 gene family as a putative novel PD-(D/E)XK endonuclease [14]. This superfamily of restriction endonuclease-like fold proteins includes several restriction endonucleases (e.g. EcoRI, EcoRII, BamHI, BglI, Cfr10I, NaeI), DNA repair enzymes (MutH and Vsr), Holliday junction resolvases (Hjc and Hje) and other nucleotide-cleaving enzymes [15]. However, no endonuclease activity has been demonstrated experimentally for any of the UL24 homologues.

Global mutational analysis of the HCMV genome classified UL76 as an augmenting gene for viral replication [16], recently demonstrated to be involved in the regulation of the UL77 gene expression. Since UL77 is essential for viral replication, its regulation by UL76 may be important for efficient HCMV replication [17]. Expression of UL76 also induces cell cycle arrest at G2/M phase by inhibition of the mitotic Cdc2-cyclin B complex. Interestingly, this effect on the cell cycle is conserved in the UL24 human homologues representatives of the alpha, beta and gamma-subfamilies and the murine homologue from MHV-68 (ORF20) [18], [19]. The precise mechanism of cell cycle arrest induced by UL24 homologues remain to be clarified, but a recent report showed that HCMV UL76 induces chromosomal aberrations and DNA damage [20].

Here we identify a new function of UL76, the induction of IL-8 expression, mediated by the ATM kinase and activation of the NF-kB pathway. Thus, activation of NF-kB by UL76 results from induction of the DNA damage response, similar to genotoxic drugs. Importantly, induction of IL-8 by HCMV is significantly reduced in the absence of ATM or in normal fibroblasts infected with a HCMV UL76 deletion mutant. Thus, viral infection induces IL-8 in a similar manner to the UL76 gene alone and UL76 is essential for maximal activation of IL-8 by HCMV.

## Results

### UL76 induces expression of IL-8

**Figure 1:**
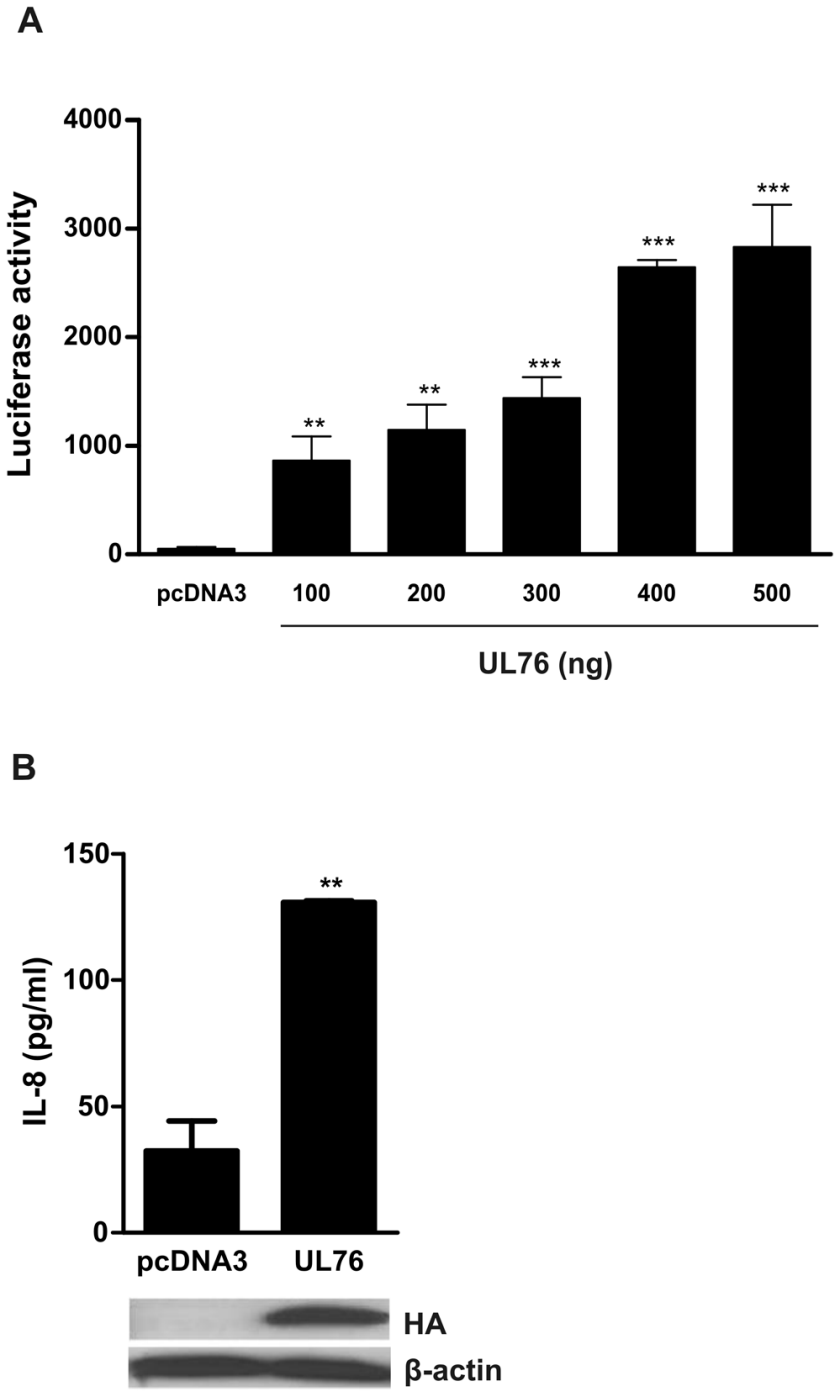
UL76 induces expression of IL-8. A) Luciferase activity was measured in 293T cells co-transfected with pcDNA3 or pcDNA3HA-UL76, IL-8 luciferase reporter and β-Galactosidase plasmid at 28 h–30 h post-transfection. Luciferase activity was normalized to β-Galactosidase activity as a control for transfection efficiency. Data are expressed as means ± SD of triplicate wells from one of three similar experiments. Statistical significance is represented as ** *p<0.01*; *** *p<0.001*. B) IL-8 concentration in supernatants of 293T cells transfected with control plasmid or pcDNA3HA-UL76 was determined 48 h post-transfection by ELISA. Data are expressed as means ± SD of duplicate wells from one of three similar experiments. ** Statistically significant as compared with control vector-expressing cells *(p<0.01)*. Expression of HA-tagged UL76 protein was confirmed by western blot (below) using an anti-HA HRP-conjugated antibody and β-actin detection was used as loading control.

The effect of UL76 on the activation of IL-8 transcription was demonstrated using a luciferase reporter construct containing the IL-8 promoter sequence. Transfection of a UL76 expression plasmid significantly activated transcription of IL-8 promoter in a dose-dependent manner (Fig. 1A). Furthermore, cells expressing UL76 were demonstrated to secrete significantly higher levels of IL-8 as compared to the control vector (*p<0.01*) (Fig. 1B). In conclusion, UL76 induces IL-8 expression at both the level of transcriptional activation and protein secretion.

### Induction of IL-8 by UL76 is NF-kB-dependent

**Figure 2:**
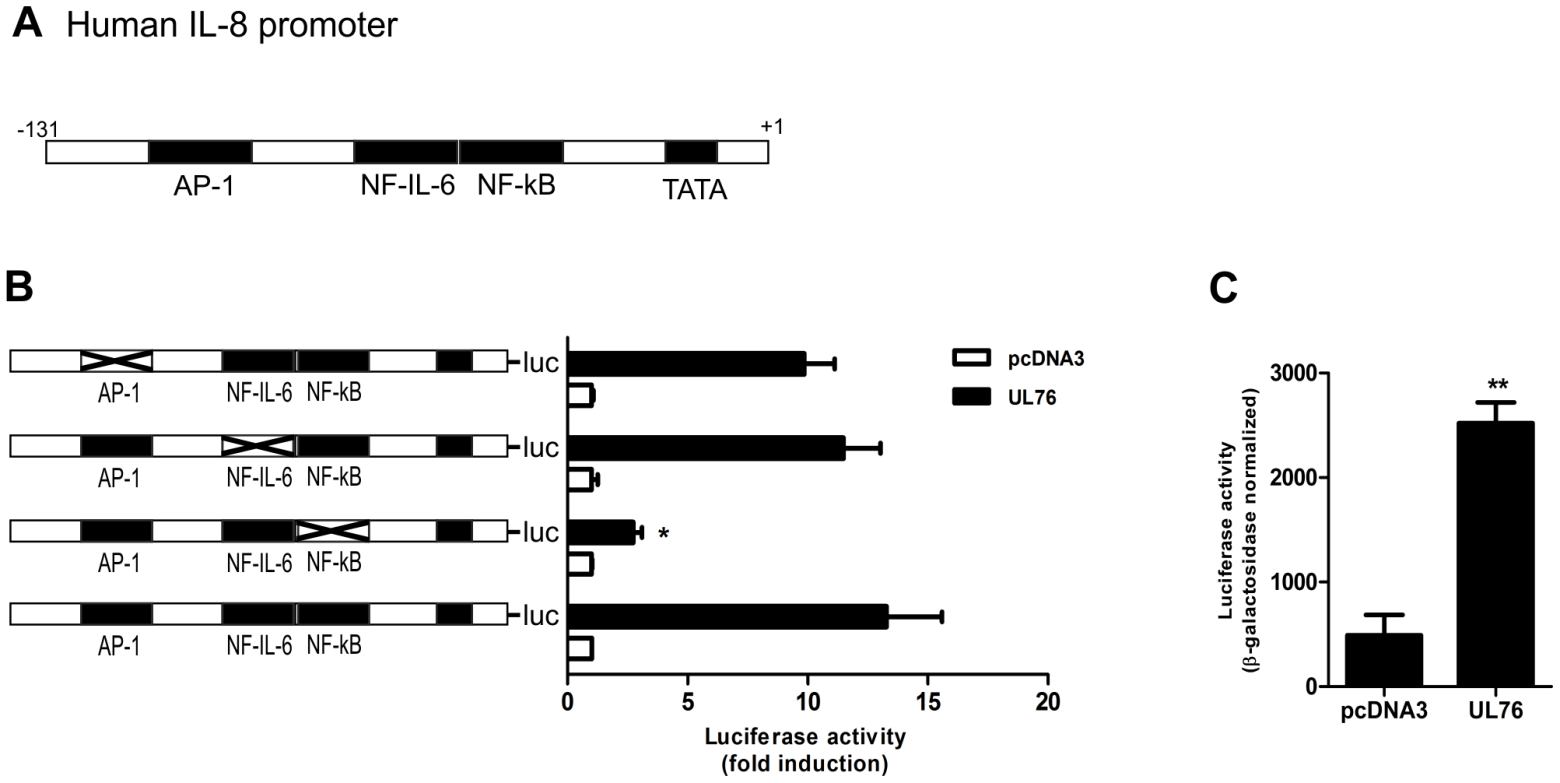
Induction of IL-8 by UL76 is NF-kB-dependent. A) Schematic representation of 5′-flanking region of human IL-8 promoter from −131 to +1 base pair, demonstrating locations of defined binding sites for NF-KB, NF-IL-6 and AP-1 transcription factors. B) Luciferase activity was measured in 293T cells co-transfected with pcDNA3 or pcDNA3HA-UL76 and wild-type IL-8 promoter or each one of the deletion mutant luciferase reporter constructs. Luciferase activity was normalized to β-Galactosidase activity as control of transfection efficiency. Data is expressed as fold induction of control vector and is representative of three similar experiments. The x indicates the mutation on AP-1, NF-IL-6 and NF-kβ binding sites within the IL-8 promoter. Statistical significance compared to IL-8 wild type promoter is represented as * *p<0.05*. C) Luciferase activity was determined in 293T cells co-transfected with pcDNA3 or pcDNA3HA-UL76 and the NF-kB luciferase reporter as previously described. Data are expressed as means ± SD of triplicate wells from one of three similar experiments. Statistical significance is represented as ** *p<0.01*.

Expression of IL-8 is tightly regulated at the transcriptional level. The sequence of nucleotides -1 to -131 in the proximal promoter region of IL-8 gene is essential for its transcription regulation and contains binding sites for NF-kB, AP-1 and NF-IL-6 transcription factors (Fig. 2A) [4]. To determine the mechanism of induction of IL-8 by UL76, the luciferase activity of wild type IL-8 luciferase reporter was compared with its mutant derivatives containing a mutation in each of the three transcription factor binding sites. There was no significant difference in luciferase activity in response to co-transfection with UL76 when AP-1 or NF-IL-6 binding sites were mutated in the luciferase reporter construct, whereas IL-8 transcriptional activation was drastically reduced in the absence of the NF-kB binding site, indicating a critical role for the NF-kB pathway in the UL76-mediated induction of IL-8 (Fig. 2B). Consistent with the previous results, expression of UL76 significantly activated an NF-kB responsive promoter (Fig. 2C).

**Figure 3:**
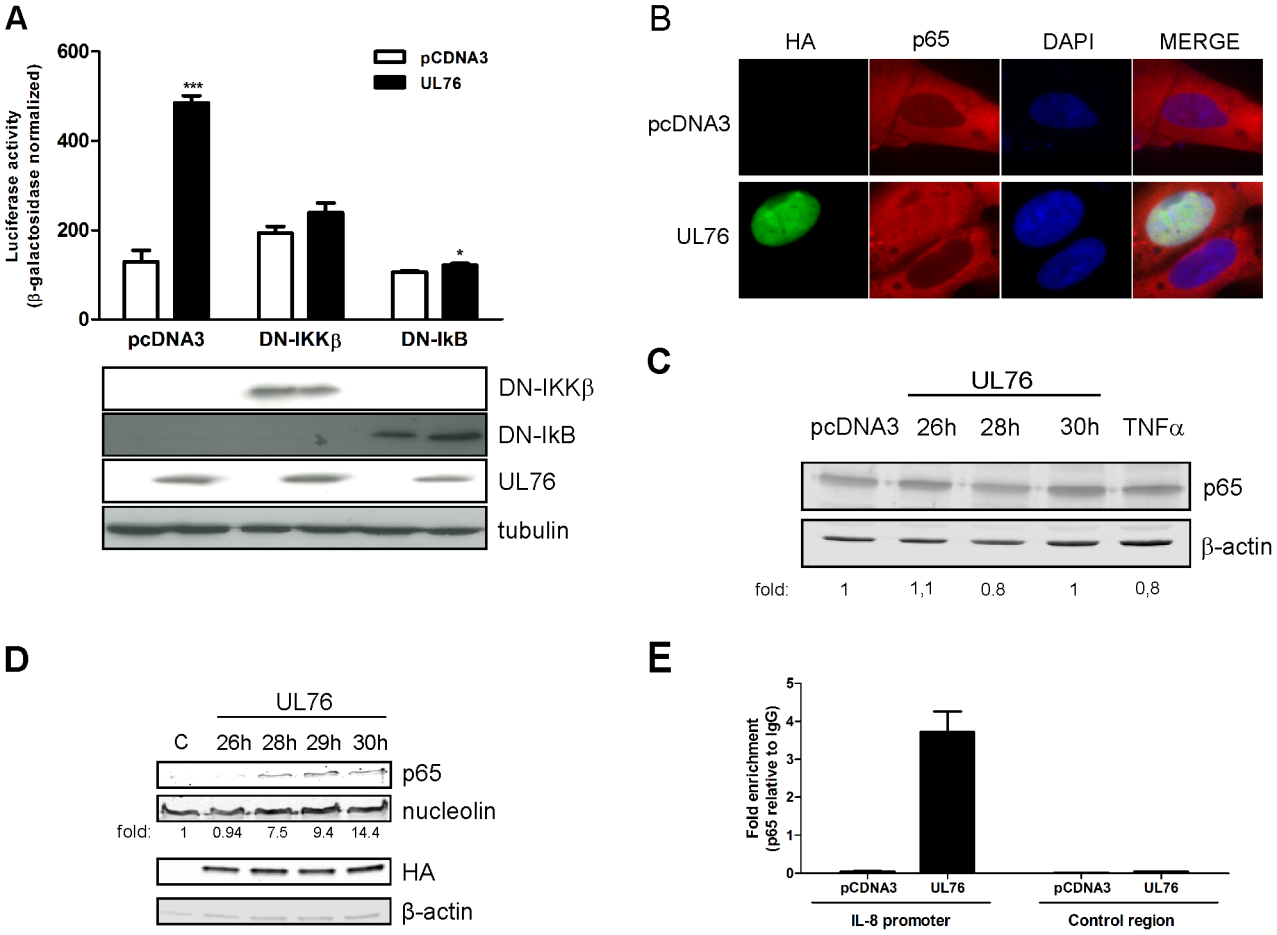
UL76 activates the NF-kB canonical pathway. A) Luciferase activity was determined in 293T cells co-transfected with pcDNA3 or pcDNA3HA-UL76, IL-8 luciferase reporter and either dominant negative IKKβ or IkBα (S32/36A) plasmids. Values of luciferase activity were normalized to β-Galactosidase activity. Data are expressed as means ± SD of triplicate wells from one of three similar experiments. Expression of UL76 and dominant negatives IKKβ or IkBα (S32/36A) was confirmed by western blot (below) using an anti-HA HRP-conjugated antibody. Detection of tubulin was used as loading control. B) Indirect immunofluorescence was performed on HFF cells transfected with pcDNA3HA-UL76 or control plasmid using an anti-p65 antibody and anti-mouse Texas Red conjugated secondary antibody. UL76 expression was detected using an anti-HA-FITC conjugated antibody and DAPI staining was used to define nuclei. Images were acquired with a 100× objective. C) Expression of p65 in HFF cells transfected with pcDNA3HA-UL76 or control plasmid was evaluated by western blot at the indicated time points. Detection of β-actin was used as loading control. As positive control cells were stimulated with TNFα. Levels of p65 relative to the levels of β-actin in the corresponding extracts were quantified by densitometry analysis and the fold levels relative to the control vector transfected cells are indicated below. D) 293T cells transfected with pcDNA3HA-UL76 or control plasmid were lysed for nuclear extraction at the indicated time points post-transfection. Nuclear extracts were immunoblotted with anti-p65, anti-β-actin and anti-nucleolin antibodies. Expression of UL76 was detected using an anti-HA antibody. Levels of p65 relative to the levels of nucleolin in the corresponding nuclear extracts were quantified by densitometry analysis. The fold levels relative to the control vector transfected cells are indicated below. E) Binding of NF-kB p65 subunit to the IL-8 promoter sequence (−121 to +61) or to a control region (−1042 to −826) in 293T cells expressing pcDNA3HA-UL76 or control plasmid was analyzed by ChIP assay at 30 h post-transfection using an anti-p65 antibody. Immunoprecipitated DNA was analyzed by quantitative real-time PCR. Data are expressed as fold enrichment relative to an unrelated IgG antibody used as control to nonspecific immunocomplexes.

To further characterize the activation of the NF-kB pathway by UL76, we used a catalytically inactive mutant IKKβ and a mutant IkBα (IkBαS32/36A) in which the two critical serine residues were mutated to alanine, thus no longer permitting its phosphorylation and degradation. Both constructs function as dominant negatives, inhibiting the activity of cellular wild type IKKβ and IkBα, respectively. Co-transfection of each dominant negative with IL-8 luciferase reporter and UL76 expression plasmid or control vector resulted in a reduced induction of IL-8 in cells expressing UL76 (Fig. 3A).

After IkBα degradation by the proteosome, dimers of NF-kB subunits translocate to the nucleus where they bind to the target gene promoter region and activate transcription [9]. Thus, we evaluated the effects of UL76 expression on the subcellular localization of the NF-kB p65 subunit by immunofluorescence. As shown in Figure 3B, p65 was localized in the nucleus of the UL76-transfected HFF cells, in contrast to its cytoplasmic localization in control cells. This effect was not due to an increase in the expression of p65 (Fig. 3C). Furthermore, similarly immunoblotting of 293T nuclear extracts with anti-p65 antibody revealed an accumulation of p65 in the nucleus of cells expressing UL76 (Fig. 3D). This accumulation was specific, as can be seen from the constant levels of the nucleolin expression in the loading control. Consistent with these results, chromatin immunoprecipitation (ChIP) analysis demonstrated that expression of UL76 leads to NF-kB p65 binding to the IL-8 promoter (Fig. 3E).

In summary, IL-8 induction by UL76 requires a functional IKKβ and the degradation of IkBα to promote translocation of p65 subunit to the nucleus where it activates IL-8 transcription.

### UL76 induces IL-8 expression through activation of the DNA Damage

**Figure 4:**
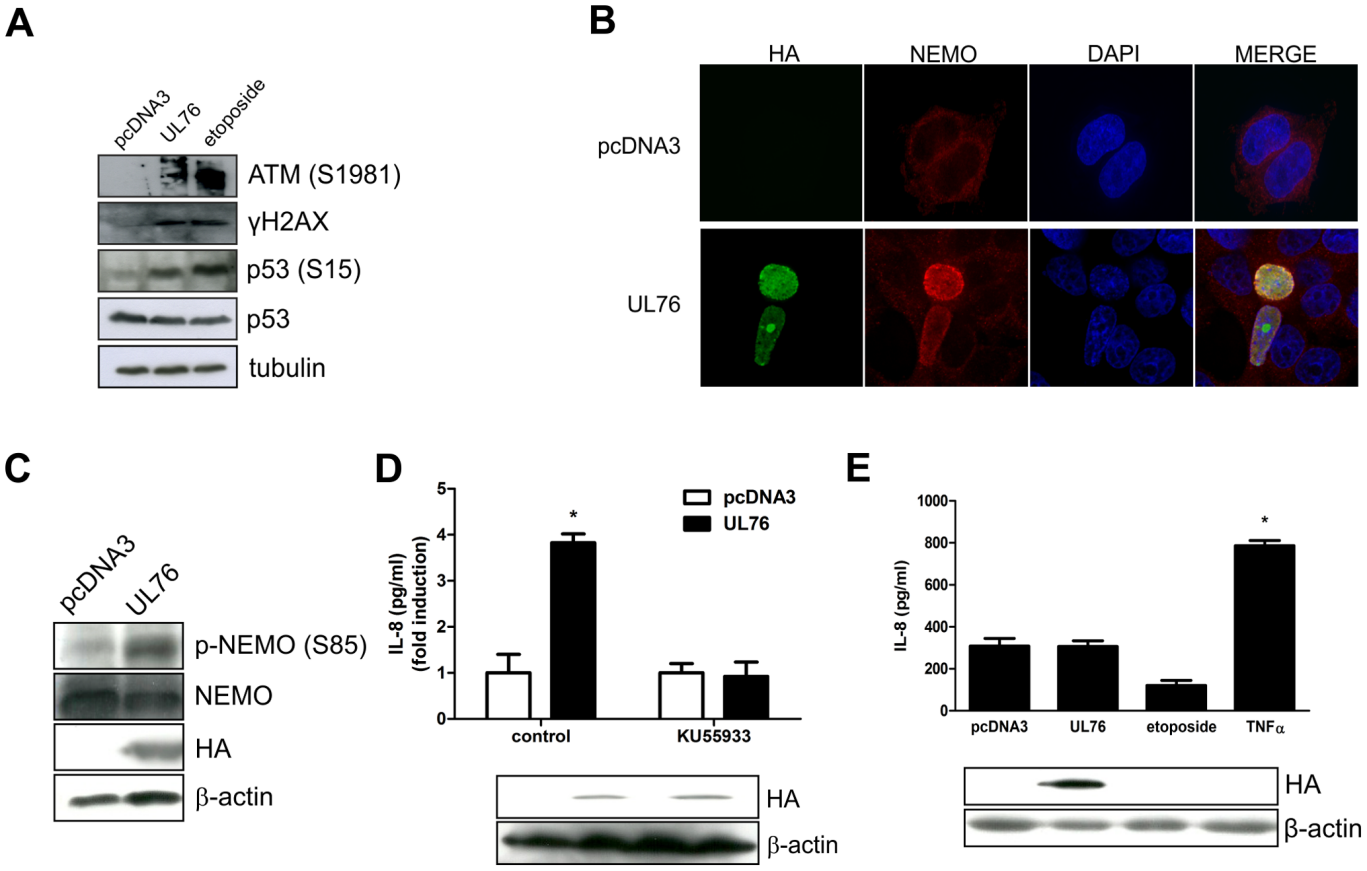
Induction of IL-8 by UL76 is mediated by activation of the DNA Damage response. A) 293T cells expressing UL76 were lysed 48 h post-transfection and the levels of ATM(S1981), γH2AX, p53(S15) and p53 were detected by western blot. Expression of UL76 was confirmed using an anti-HA-HPR conjugated antibody and detection of tubulin was used as loading control. B) Subcellular localization of NEMO in 293T cells transfected with pcDNA3HA-UL76 or control plasmid was determined by indirect immunofluorescence using an anti-NEMO antibody. UL76 expression was detected using HA-FITC conjugated antibody and DAPI staining was used to define nuclei. Images were acquired with a 100× objective. C) Phosphorylation of NEMO at serine 85 in 293T cells transfected with pcDNA3HA-UL76 or control plasmid was detected at 24 h post-transfection by western blot using a specific anti-p-NEMO(S85) or NEMO antibody. Expression of UL76 was confirmed using an anti-HA-HPR conjugated antibody and detection of β-actin was used as loading control. D) IL-8 concentration in supernatants of 293T cells expressing UL76 or control plasmid in the presence or absence of ATM inhibitor, KU55933 (10 µM), was determined 48 h post-transfection by ELISA. Data are expressed as fold induction to control vector (means ± SD) from one of three similar experiments. Expression of UL76 was confirmed by western blot (below) and detection of β-actin was used as loading control. E) IL-8 concentration in supernatants of *ATM -/-* cells expressing UL76 or control plasmid was determined 48 h post-transfection by ELISA. Cells were stimulated for 5 h with etoposide (10 µM) or TNFα (10 ng/ml) as controls. Data are expressed as means ± SD of duplicate wells from one of three similar experiments. Expression of UL76 was confirmed by western blot (below) and detection of β-actin was used as loading control.

Expression of UL76 results in an increased number of double stranded DNA breaks and phosphorylation of γH2AX, indicating activation of the DNA Damage response [20]. Consistent with these results, expression of UL76 results in activation of ATM and consequent phosphorylation of p53 and H2AX proteins (Fig. 4A). Recently, several studies have characterized an alternative pathway to NF-kB activation that results from DNA damage. Based on this, we hypothesized that the ability of UL76 to induce DNA damage would lead to activation of NF-kB pathway and result in the induction of IL-8 expression. A characteristic feature of NF-kB pathway activation by genotoxic stress is the accumulation of IKKγ/NEMO in the nucleus where a series of post-translational modifications occurs [21]. The nuclear post-translational modifications of NEMO that are critical for NF-κB activation following genotoxic stress include ATM-independent sumoylation and ATM-dependent phosphorylation at serine 85 followed by monoubiquitination. Consistent with this mechanism, immunostaining using an anti-NEMO antibody revealed increasing amounts of nuclear NEMO in cells expressing the UL76-HA tagged protein (Fig. 4B). Furthermore, immunoblotting of similarly transfected cells with a specific antibody to NEMO(S85) phosphorylation demonstrated that expression of UL76 induces phosphorylation of NEMO as previously observed after genotoxic stress (Fig. 4C).

To evaluate the impact of the ATM kinase in IL-8 induction by UL76, we used two different approaches: a specific ATM inhibitor, KU55933, and a human fibroblast cell line deficient in ATM. The amount of IL-8 secreted by 293T cells expressing UL76, or the control plasmid, in the presence or absence of KU55933 was determined by ELISA. Inhibition of ATM by KU55933 blocked the UL76-induced IL-8 secretion (Fig. 4D). Similarly, IL-8 concentration was determined in supernatants of *ATM -/-* cells expressing UL76 or the control plasmid. As shown in Figure 4E, UL76 or etoposide, a genotoxic drug, are unable to induce IL-8 in the absence of ATM. Although UL76 expression leads to higher levels of IL-8 than etoposide stimulation, there is no increase in IL-8 secretion when compared to control vector, so this basal induction is possibly due to transfection. Moreover, this result is not due to the incapacity of the cell line to produce IL-8 since stimulation with TNFα, a membrane receptor-triggering NF-kB canonical pathway independent of ATM, is still capable of inducing IL-8 secretion (Fig. 4E). Expression of UL76 was not affected in the *ATM -/-* cell line or in 293T cells cultured in the presence of the ATM inhibitor as confirmed by western blot (Fig. 4D,E). In summary, these results indicate that activation of NF-kB pathway and consequent IL-8 induction by UL76 are ATM-dependent and result from activation of DNA damage.

### Mutation of the putative UL76 endonuclease motifs reduces induction of IL-8

**Figure 5:**
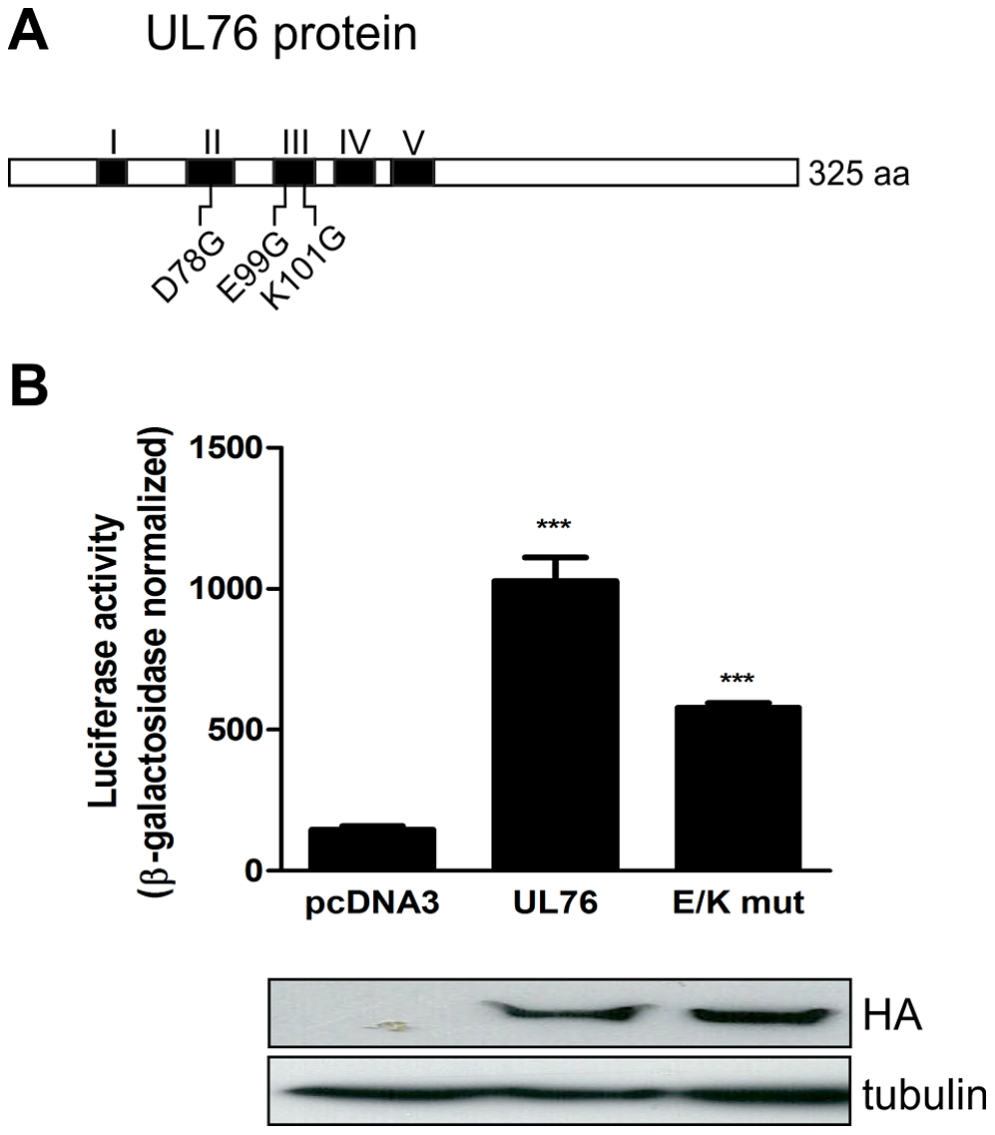
Mutation of the putative endonuclease motifs of UL76 reduced induction of IL-8. A) Schematic representation of UL76 protein. Conserved domains in UL24 gene family is represented in black and numbered from I–V. The mutations of the conserved PD-(D/E)XK signature amino acids in the UL76 protein are indicated below. B) Luciferase activity was measured in 293T cells co-transfected with pcDNA3, pcDNA3HA-UL76 or pcDNA3HA-E/K mut, IL-8 luciferase reporter and β-Galactosidase plasmid at 28 h–30 h post-transfection. Luciferase activity was normalized to β-Galactosidase activity as a control of transfection efficiency. Data are expressed as means ± SD of triplicate wells from one of three similar experiments. Statistical significance is represented as *** *p<0.001*.

There is clear evidence that UL76 activates the DNA damage response, however, the mechanism employed by UL76 for this activation is still unknown. The prediction that the UL24 gene family encodes a novel PD-(D/E)XK endonuclease is a possible explanation [14]. Comparison of UL24 gene family sequences identified the three conserved PD-(D/E)XK signature amino acids of the endonuclease motif which are conserved in all homologues (Fig. 5A) [14]. A UL76 gene with these three critical amino acids mutated was constructed and used to determine the impact of the putative endonuclease activity on IL-8 induction. Levels of IL-8 secreted by cells expressing the mutant UL76 gene were reduced when compared to wild type UL76 gene; however, they were still significantly higher than control vector-expressing cells (Fig. 5B). These results indicate that the putative endonuclease activity is not essential for the induction of IL-8.

### Induction of IL-8 by HCMV is reduced in the absence of UL76

**Figure 6:**
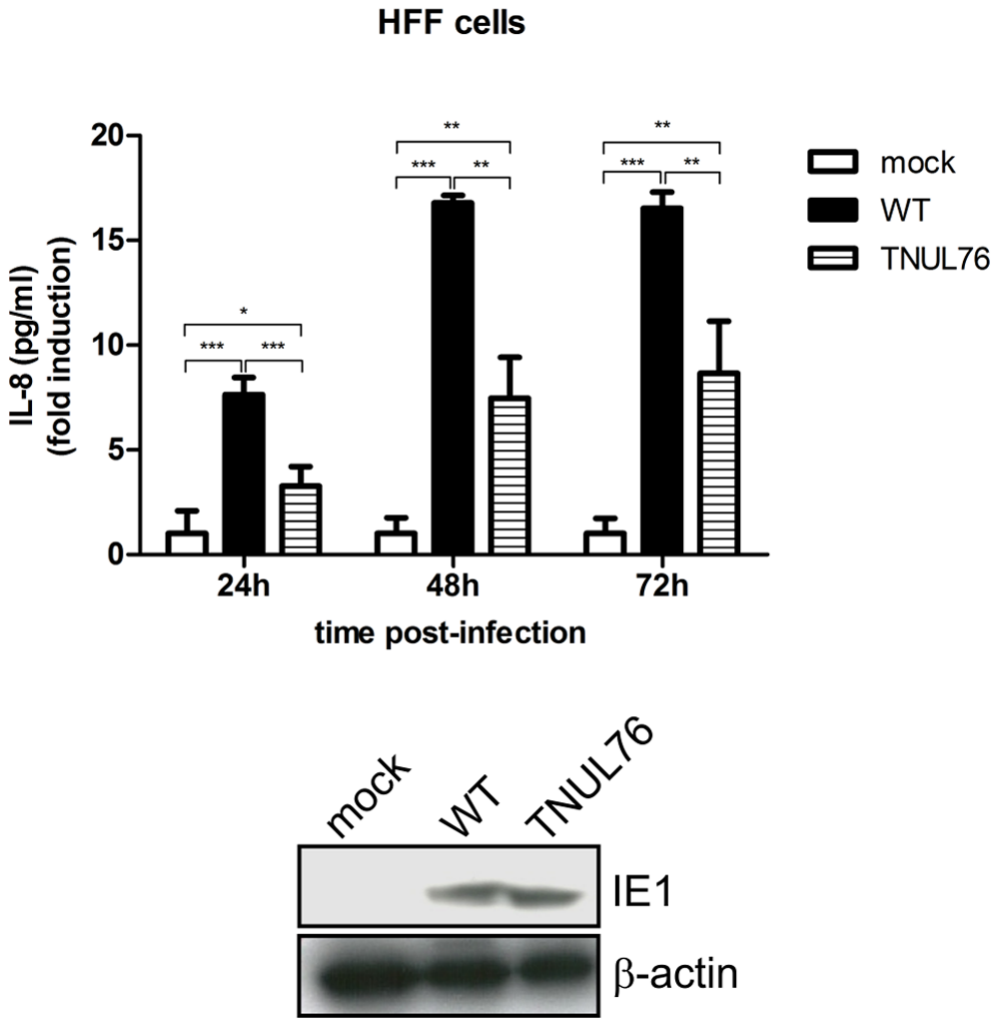
Requirement of UL76 for optimal IL-8 induction by HCMV. A) HFF cells were infected with HCMV AD169 or UL76 mutant (TNUL76) at a MOI of 3. Supernatants were collected at 24 h, 48 h and 72 h post-infection and secreted IL-8 concentration was determined by ELISA. Data are expressed as means ± SD of fold induction to mock-infected cells and are representative of three similar experiments. Cells were lysed at 72 h post-infection and viral infection was confirmed by western blot (below) using an anti-IE1 antibody for the CMV viral protein and detection of tubulin as loading control.

In order to evaluate the impact of UL76 on the up-regulation of IL-8 in the context of HCMV infection, we used a previously described UL76 transposon mutant HCMV [16]. Supernatants from human fibroblasts infected with wild type HCMV AD169 BAC or UL76 mutant virus (TNUL76) were collected at the indicated time points and secreted IL-8 was determined by ELISA. Consistent with previous studies [3], [22], HCMV infection resulted in high levels of IL-8 secretion during the course of the experiment (Fig. 6A). Induction of IL-8 in cells infected with the UL76 mutant virus, however, was significantly reduced. At each time point, the amount of IL-8 secreted by cells infected with UL76 mutant virus (TNUL76) was reduced by 42–52% compared with wild type HCMV. Equal infection by both viruses was confirmed by levels of the HCMV immediate-early 1 (IE1) protein. Thus, UL76 is essential for optimal induction of IL-8 by HCMV. There was, however, no difference in the phosphorylation of ATM, NEMO and IkB proteins in cells infected with HCMV wild type compared to UL76-deficient virus (data not shown).

As the mutation of UL76 significantly increases the level of UL77 protein expression [17], the effect of UL77 in the expression of IL-8 was evaluated by ELISA. In contrast to UL76-transfected cells, there is no induction of IL-8 in cells expressing UL77. Moreover, UL77 has no inhibitory effect in the induction of IL-8 by different stimuli (Fig. S1). Overall, these results demonstrate that UL77 is not able to modulate IL-8 expression and thus, the reduction of IL-8 levels in cells infected with the UL76 mutant virus (TNUL76) is not due to the regulation of UL77 expression by UL76.

### ATM is required for induction of IL-8 by HCMV

**Figure 7:**
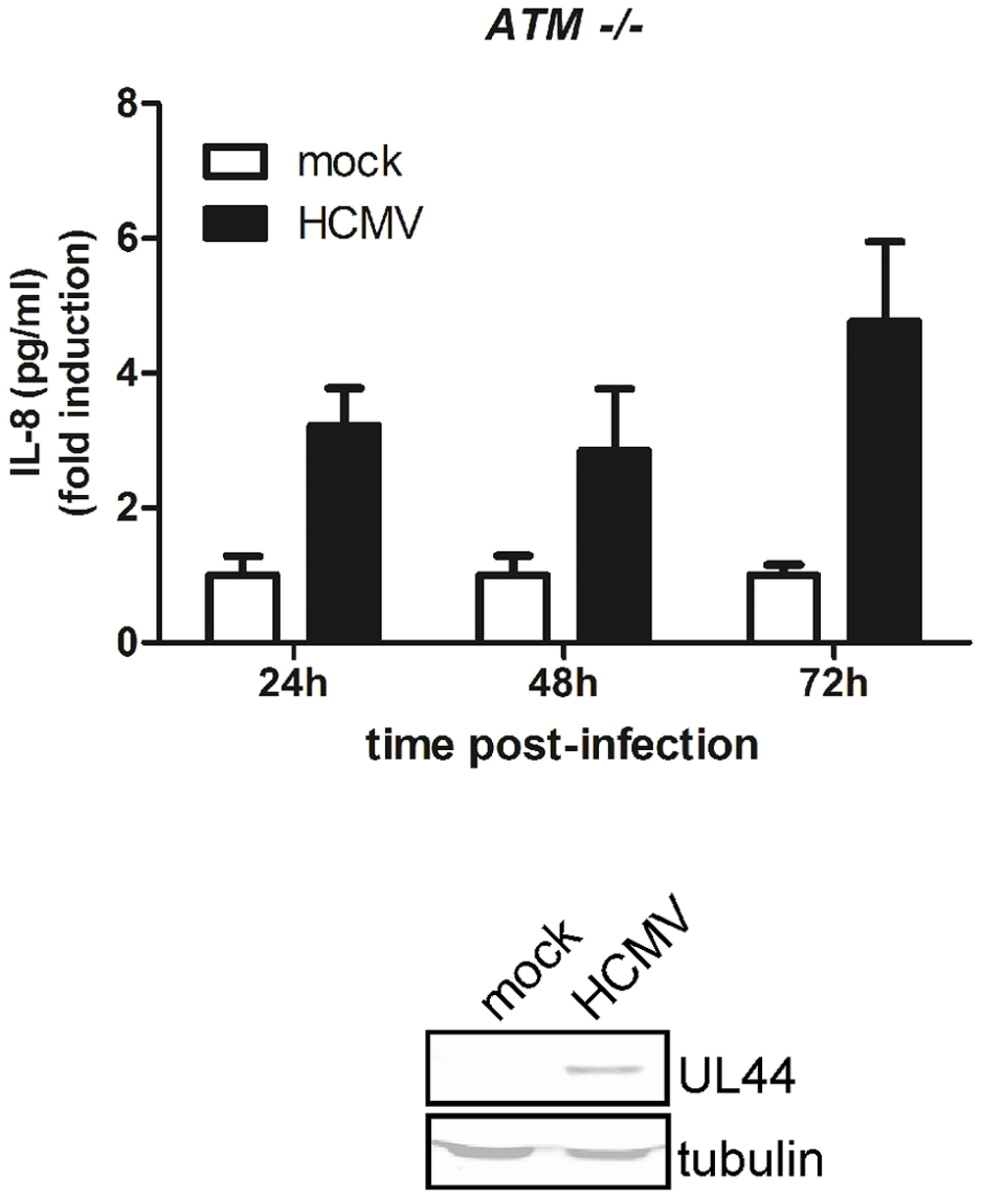
Impact of ATM on the induction of IL-8 by HCMV. Primary fibroblasts *ATM -/-* cells (GM01588) were infected with HCMV wild type at a MOI of 3. Supernatants were collected at 24 h, 48 h and 72 h post-infection and IL-8 concentration was determined by ELISA. Data are expressed as means ± SD of fold induction to mock-infected cells and are representative of three similar experiments. Cells were lysed at 72 h post-infection and viral infection was confirmed by western blot (below) using an anti-UL44 antibody for the HCMV viral protein and detection of β-actin as loading control.

A previous deletion mutant analysis of the IL-8 promoter in monocytic cells has shown that AP-1 and NF-kB transcription factors were required for optimal induction of IL-8 by HCMV [22]. The precise mechanism used by HCMV to induce IL-8 expression, however, is still not clear. As UL76 is required for maximal IL-8 induction by HCMV (Fig. 6) and HCMV infection activates ATM [23], [24], we hypothesized that ATM would also have a role in IL-8 up-regulation during viral infection. To test this hypothesis, a primary fibroblast *ATM -/-* cell line was infected with HCMV AD169 BAC virus. Supernatants were collected at the indicated time points and IL-8 concentration was determined by ELISA. When compared to normal human fibroblasts (Fig. 6), the amount of secreted IL-8 was significantly reduced in the HCMV infected *ATM -/-* cells (16,79 vs 3,63 fold induction) (Fig. 7). Similar results were obtained with a transformed *ATM -/-* fibroblast cell line (data not shown). Infection with HCMV was confirmed by the presence of the viral protein UL44 (Fig. 7, below). Similar to wild type HCMV, *ATM -/-* cells infected with the UL76 deficient virus (TNUL76) secreted lower levels of IL-8 compared to HFF infected cells. The levels of IL-8 of cells infected with TNUL76 were, however, even lower than the observed in *ATM -/-* cells infected with wild type virus (Fig. S2). Collectively, these results indicate that during HCMV infection, UL76, and possibly other gene(s), induces IL-8 expression, at least in part, through activation of ATM.

## Discussion

The UL24 gene family is one of the approximately 40 core genes that are conserved in all three herpesviruses subfamilies and the only one which still has no assigned function [13]. Previously, functional assays demonstrated that all homologues of UL24 gene family induce cell cycle arrest [18], [19]. This work identifies another, and at first sight, apparently unrelated function of the UL24 homologue from HCMV (UL76), the induction of the expression of IL-8. Further exploration of the mechanism, however, suggests that both activities may result from viral activation of the DNA Damage response.

Deletion mutant analysis of the IL-8 promoter demonstrated that UL76 up-regulates IL-8 expression through activation of NF-kB pathway requiring a functional IKKβ and degradation of the IkB protein. Moreover, expression of UL76 resulted in the translocation of the NF-kB p65 subunit to the nucleus and its binding to the IL-8 promoter. These events, characteristic of the canonical NF-kB pathway, typically occur in the cytoplasm and are usually activated by membrane-receptor stimulation [9]. Thus, the exact mechanism of how UL76 activates the NF-kB pathway is an interesting paradox as UL76 is a nuclear protein.

In recent years an alternative mechanism of activation of NF-kB pathway triggered by genotoxic stress has been described. In contrast to inflammatory stimuli such as TNFα or IL-1β, the signal originates in the nucleus [10]. Since it has been shown that UL76 is able to induce double strand breaks, and consequently activate DNA damage [20], we hypothesized that UL76 might induce IL-8 expression as result of the DNA damage response. Indeed, and similar to the effect of genotoxic drugs such as etoposide, expression of UL76 resulted in an accumulation of nuclear NEMO and its activation (phosphorylation at serine 85). These findings suggest that the ATM kinase might play a role in the IL-8 induction by UL76 and thus, the predicted role of ATM was demonstrated by two strategies, one using a specific ATM inhibitor and the other employing an ATM knockout cell line. Abrogation of UL76-mediated induction of IL-8 occurred with both approaches. These observations indicate that induction of IL-8 by UL76 originates in the nucleus as a result of the DNA Damage response.

The exact mechanism of activation of the DNA Damage response induced by UL76 is still not clear. A promising clue that we pursued was the identification of conserved putative PD-(D/E)XK endonuclease motifs in the UL24 gene family [14]. Its conservation suggests a critical role in the function of this gene family, thus, the DNA damage activation by UL76 could be a direct effect of its endonuclease activity. Mutation of the three predicted endonuclease signature amino acids was, however, inconclusive as it resulted in a reduction rather than an abolition of IL-8 induction. It is possible that the observed reduction may be related with an unknown function of these conserved domains rather than loss of a putative endonuclease activity. In fact, the bioinformatically predicted endonuclease motif might not be related to a functional endonuclease activity, as no endonuclease activity has been demonstrated experimentally for any of the UL24 homologues.

Importantly, UL76 has a critical role in the up-regulation of IL-8 during HCMV infection as demonstrated by the significant reduction of secreted IL-8 in cells infected with an UL76 deletion mutant virus. This result is particularly important as IL-8 enhances HCMV replication and contributes to the efficient viral dissemination by neutrophils [2], [3]. The only HCMV gene that has been described as an activator of IL-8 is the Immediate Early 1 gene (IE1) [22]. Thus, the incomplete inhibition of IL-8 secretion by HCMV observed in the absence of UL76 may be due to the effect of IE1 gene. The existence of other gene(s) that may also contribute for HCMV-induced IL-8 expression, in addition to IE1 and UL76, is not excluded. Experiments to observe a similar impact of UL76 in the signaling pathway at the level of virus infected cells were negative, possibly due to alternative virus strategies activating these proteins. We emphasize, however, that the key observation is the diminished expression of IL-8 induced by the UL76 deficient virus, which clearly demonstrates a role for UL76 in the up-regulation of IL-8 in HCMV infected cells.

The relevance of IL-8 in HCMV life cycle is emphasized by the fact that the HCMV UL146 gene encodes a homologue to CXC chemokines such as IL-8 (vCXCL1), which functions as a selective agonist for CXCR2 and, with lower affinity, for CXCR1 [25], [26]. The IL-8 production observed in cells infected with wild type HCMV or UL76 mutant virus, however, is independent of the presence of the viral CXCL1, as this gene is deleted from the HCMV AD169 strain used in this work [16].

Induction of IL-8 expression by HCMV requires activation of the NF-kB pathway [22]. Thus, one objective of this work was to elucidate the mechanism of NF-kB activation by HCMV that leads to IL-8 production. Here we demonstrate that ATM also has a critical role in the induction of IL-8 by HCMV as infection of *ATM -/-* cells with wild type HCMV resulted in considerably lower levels of secreted IL-8 compared to the similar infection of normal human fibroblasts. On the other hand, the incomplete inhibition of IL-8 expression in *ATM-/-* cells infected with HCMV suggests that other NF-kB pathways are involved. It is possible that these are not redundant effects, but activation of different NF-kB pathways, possibly through different viral proteins, may be necessary for the induction of optimal levels of IL-8 by infected cells that will be beneficial for HCMV replication as previously reported [2].

It may be significant that a major reduction in viral replication is observed when normal cells are infected with a UL76 deficient virus [16] or when ATM deficient cells are infected with HCMV wild type virus [27]. It is possible that this defect in viral replication is associated with the reduced IL-8 levels observed in cells infected with UL76 deficient HCMV or in ATM deficient cell infected with wild type HCMV. Supporting this hypothesis is the fact that IL-8 enhances HCMV replication [2].

In summary, the non-homologous UL76 gene of HCMV has not only evolved for manipulation of the host cell cycle, but also activates expression of the pro-inflammatory chemokine IL-8. Both of these activities appear to depend on activation of pathways triggered as a result of the DNA Damage response and may favor propagation of the virus. The fact that, in recent years, several viruses have been demonstrated to activate the DNA Damage response raised new important questions. It is not known if this activation results from recognition of DNA damage or if it is due to the recruitment of DNA repair proteins observed during viral infections such as HCMV. Furthermore, it is not completely understood how the activation of DNA Damage pathway is beneficial for viral replication. Our present work establishes a new role of the induction of DNA Damage response in the context of viral infection that may help to elucidate some of these questions, as it demonstrates how viruses exploit the complex crosstalk that occur between different cell signaling pathways.

## Materials and Methods

### Cell lines

Human embryonic kidney 293T cells were cultured in 5% CO_2_ in Dulbecco's Modified Eagle's Medium (Gibco) supplemented with 10% fetal calf serum (Gibco) at 37°C. Human foreskin fibroblasts (HFF) (obtained from European Collection of Cell Cultures), a transformed (GM09607) and a primary (GM01588) A-T human fibroblast cell lines (obtained from the Coriell Institute for Medical Research) were cultured in Minimum Essential Medium with Earle's salts supplemented with 10% fetal calf serum (Gibco).

### Plasmids

The UL76 and UL77 gene from HCMV AD169 were cloned into pcDNA3 plasmid fused in frame with an amino-terminal influenza haemaglutinin peptide (HA) tag. The three putative endonuclease amino acids in the UL76 gene were mutated to glycine (pcDNA3HA-E/K mut plasmid) according to the Directed Mutagenesis kit protocol (Stratagene). The luciferase reporter constructs containing human IL-8 promoter (-131) or a mutation in the NF-kB, AP-1 or NF-IL-6 binding site were a gift from Dr Naofumi Mukaida and have been described before [22]. The reporter plasmid for NF-κB [p(PRD2)5tkΔ(-39)lucter] was a gift from Dr Steve Goodbourn. Dominant negative mutants of IKKβ and IkBα (S32/36A) plasmids containing an HA tag, were obtained from Dr Michael Karin [28] and Dr Dean Ballard [29], respectively. The pCMVβ plasmid contains a β-galactosidase gene under the control of human cytomegalovirus immediate early promoter.

### Virus stock preparation

The HCMV laboratory strain AD169 bacterial artificial chromosome (BAC) DNA was obtained from Dr Ulrich Koszinowski. The UL76 mutant virus (TNUL76), a gift from Dr Thomas Shenk, was generated by site-directed transposon mutagenesis of HCMV AD169 BAC and has been previously described [16]. Wild type or UL76 mutant virus BAC DNA were transfected in HFF cells by electroporation. Supernatants of transfected cells were collected and used for virus stock production. To prepare virus stocks of wild type AD169 BAC virus and TNUL76 mutant virus, HFF cells were infected at a multiplicity of infection (MOI) of 0.01. After virus adsorption for one hour, infected cells were cultured at 37°C and medium was collected every three days. Pre-cleared supernatants were centrifuged two hours at 12000 rpm at room temperature. Virus aliquots were stored at −80°C. Virus stock titers were determined by plaque assay. Briefly, HFF cells were cultured with 10-fold dilutions of virus suspension and allowed to absorb for 1 h. Cells were then cultured with complete medium containing 10% carboxymethylcellulose (CMC) for 10–15 days. Cellular monolayers were fixed in 4% paraformaldehyde and stained with 0.1% toluidine blue. Quantification of the viral plaques was performed using a dissecting microscope.

### Luciferase reporter assays

293T cells were co-transfected in triplicate with 100 ng of IL-8 luciferase reporter plasmid or luciferase reporter constructs containing mutations in the IL-8 promoter (ΔNF-kB, ΔAP-1 and ΔNF-IL-6), 25 ng of β-galactosidase internal control plasmid (pCMVβ) and 300 ng of pcDNA3 or pcDNA3HA-UL76, according to the Lipofectamine 2000 (Invitrogen) protocol. A similar transfection protocol was performed using the NF-kB luciferase reporter plasmid. Cells were lysed 28 h–30 h post-transfection and the luciferase activity was measured using the luciferase assay system (Promega) according to the manufacturer's protocol. β-galactosidase activity was measured using the Galacton-Plus kit from Tropix (Bedford, MA). The luciferase activity was normalized relative to the β-galactosidase activity of each sample as control of transfection efficiency.

### Enzyme-linked immunosorbent assay (ELISA)

Supernatants of 293T cells or *ATM -/-* (GM09607) transfected with pcDNA3 (negative control), pcDNA3HA-UL76 or pcDNA3HA-UL77 plasmids were collected at 48 h post-transfection. As control, cells were stimulated with etoposide (10 µM) (Sigma), TNFα (10 ng/ml) (Peprotech) or IL-1β (1 ng/ml) (Cell Signalling) for 5 h. The concentration of IL-8 secreted was determined using an IL-8 ELISA kit (BD Biosciences) following the manufacturer's instructions. Similarly, supernatants of HFF or *ATM -/-* (GM01588) cells infected with wild type HCMV or UL76 mutant HCMV (TNUL76) at a MOI of 3, or mock-infected, were harvested at the indicated time points and clarified by centrifugation before quantification of IL-8 by ELISA. For ATM inhibition experiments, ATM inhibitor KU55933 (10 µM) (Calbiochem) was added to cells 1 h before transfection or infection with HCMV and was maintained in the medium during the experiment. Plates were analyzed at 450 nm using a BioRad ELISA Reader (BioRad) and levels of IL-8 were determined by comparison to a standard curve.

### Immunofluorescence

The 293T or HFF cells were cultured on sterile glass coverslips and transfected with pcDNA3HA-UL76 or control pcDNA3 plasmid according to the Lipofectamine 2000 (Invitrogen) protocol. As positive control cells were stimulated with recombinant human TNFα (20 ng/ml) (Peprotech) for 30 minutes. At the indicated times post-transfection, cells were washed with PBS and fixed with 4% paraformaldehyde for 20 minutes. Fixed cells were permeabilised with PBS-0.1% Triton X-100 for 20 minutes. After washing, the cells were blocked with PBS-0.05% Tween 20 containing 5% normal goat serum for one hour. The samples were incubated with a mouse monoclonal anti-p65 (F-6) or anti-IKKγ/NEMO (B-3) antibody (Santa Cruz Biotechnology) followed by incubation with goat anti-mouse Texas Red secondary antibody (Molecular Probes) and rat monoclonal anti-HA-FITC conjugated antibody (Roche) to visualize UL76 HA-tagged protein. After incubation with DAPI, the coverslips were mounted in “Slow Fade” (Invitrogen) and images were acquired with a DeltaVision microscope (Applied Precision/Olympus).

### Nuclear extracts

293T cells were transfected with pcDNA3HA-UL76 or control pcDNA3 plasmid and nuclear extraction was performed using a Nuclear Extraction Kit according to the manufacturer's indications (Active Motif). Briefly, at the indicated time points post-transfection, cells were collected in ice-cold PBS in the presence of phosphatase inhibitors. Cytoplasmic extracts were obtained by resuspending the cells in hypotonic buffer followed by addition of detergent. After centrifugation the pelleted nuclei were lysed and nuclear proteins were solubilized in the lysis buffer supplemented with a protease inhibitor cocktail. Protein concentrations were determined by Bradford assay (Bio-Rad Laboratories).

### Western blot

Total lysates from cells transfected with pcDNA3HA-UL76 or control pcDNA3 plasmid were prepared using lysis buffer supplemented with a mixture of protease and phosphatase inhibitors (Calbiochem), for 30 minutes on ice. Protein concentrations were determined by Bradford assay (Bio-Rad Laboratories). Proteins from total or nuclear lysates were separated by sodium dodecyl sulfate polyacrylamide gel electrophoresis (SDS-PAGE) and transferred to polyvinylidene difluoride (PVDF) membrane (GE Healthcare). Membranes were blocked with 5% nonfat milk for one hour at room temperature. Primary antibodies used were: mouse monoclonal anti-p65 (F-6), mouse monoclonal anti-IKKγ/NEMO (B-3), rabbit anti-nucleolin/C23 (H-250), mouse monoclonal anti-p53, rabbit anti-ATM, mouse anti-IE1 HCMV, mouse anti-pp52 (UL44) (Santa Cruz Biotechnology), rabbit anti-IKKγ/NEMO(S85) (Assay Biotech), rabbit anti-phospho-Histone H2AX(Ser139), mouse monoclonal anti-phospho-p53(Ser15), mouse monoclonal anti-phospho-ATM(Ser1981) (Cell Signaling), mouse monoclonal anti-β-actin, anti-HA and anti-tubulin (Sigma). Mouse monoclonal anti-β-actin and rat monoclonal anti-HA horseradish peroxidase-conjugated antibodies were purchased from Sigma. IRDye 800CW anti- mouse and anti-rabbit antibodies were purchased from Li-Cor Biosciences. Immunoblots were developed by enhanced chemiluminescence detection according to the manufacturer's instructions (ECL, Thermo Scientific Pierce) or using the Odyssey Infrared Imaging System (Li-Cor; Lincoln, NE). Densiometry analysis was performed using ImageJ software or Image Studio Lite Analysis Software (Li-Cor).

### Chromatin immunoprecipitation (ChIP)

293T cells were transfected with pcDNA3HA-UL76 or control plasmid according to the Lipofectamine 2000 (Invitrogen) protocol. Thirty hours post-transfection, cells were cross-linked with 1% formaldehyde (Calbiochem) for 10 minutes at room temperature. After washing with PBS, cells were resuspended in SDS lysis buffer with protease inhibitor cocktail (Sigma) and chromatin was sheared by sonication. Immunoprecipitation was performed overnight at 4°C, using 2 µg of rabbit polyclonal anti-NF-kB p65 (A) or control IgG antibody (Santa Cruz Biotechnology). After incubation with protein G magnetic beads (Dynabeads, Invitrogen) for one hour at 4°C, immunocomplexes were washed and eluted. The cross-linking was reversed by heating at 65°C for 4 h. Chromatin-associated proteins were digested with proteinase K and DNA was purified by QIAGEN PCR purification kit following manufacturer's protocol. Immunoprecipitated DNA was quantified by real-time quantitative PCR using SYBR Green Master Mix (Applied Biosystems) and primer pair spanning the human IL-8 promoter region from −121 to +61: sense 5′-GGGCCATCAGTTGCAAATC -3′ and antisense 5′-TTCCTTCCGGTGGTTTCTTC-3′. Primers targeting the genomic region from −1042 to −826 of the IL-8 gene were used as negative control region: sense 5′-AACAGTGGCTGAACCAGAG-3′ and antisense 5′-AGGAGGGCTTCAATAGAGG-3′.

### Statistical analysis

Data were shown as mean values with standard deviation (SD). Differences between experimental groups were determined by a two-tailed Student *t* test using GraphPad Prism 5 software.

## Supporting Information

Figure S1 **UL77 has no effect in the expression of IL-8.** IL-8 concentration in supernatants of 293T cells transfected with control plasmid or pcDNA3HA-UL77 was determined 48 h post-transfection by ELISA, before and after stimulation for 5 h with TNFα (10 ng/ml) or IL-1β (1 ng/ml). Data are expressed as means ± SD of duplicate wells from one of two similar experiments. Expression of HA-tagged UL77 protein was confirmed by western blot (below) using an anti-HA antibody and β-actin detection was used as loading control.
(TIF)

Figure S2 **Impact of ATM on the induction of IL-8 by HCMV.** A) Primary fibroblasts *ATM -/-* cells (GM01588) were infected with HCMV wild type (wt) or UL76 mutant virus (TNUL76) at a MOI of 3. Supernatants were collected at 24 h, 48 h and 72 h post-infection and IL-8 concentration was determined by ELISA. Data are expressed as means ± SD of fold induction to mock-infected cells and are representative of three similar experiments. Cells were lysed at 72 h post-infection and viral infection was confirmed by western blot (below) using an anti-UL44 antibody for the CMV viral protein and detection of β-actin as loading control. B) HFF cells were transfected with ON-TARGETplus Human ATM siRNA or control siRNA (ctrl) (100 µM) 24 h prior to infection with HCMV (MOI 3). Supernatants were collected at 24 h, 48 h and 72 h post-infection and IL-8 concentration was determined by ELISA. Data are expressed as means ± SD and are representative of two similar experiments. Levels of ATM protein in cells transfected with ATM siRNA or control siRNA were determined by western blot using an anti-ATM antibody and detection of tubulin as loading control.
(TIF)

## References

[1] Mocarski ES, Shenk T, Pass RF (2007) Cytomegaloviruses. In: Knipe DM, Howley PM, editors. Fields Virology, 5th Edition: Lippincott Williams & Wilkins.

[2] Murayama T, Kuno K, Jisaki F, Obuchi M, Sakamuro D, et al (1994) Enhancement human cytomegalovirus replication in a human lung fibroblast cell line by interleukin-8. J Virol 68: 7582–7585. 7933146 10.1128/jvi.68.11.7582-7585.1994 PMC237206

[3] Craigen JL, Yong KL, Jordan NJ, MacCormac LP, Westwick J, et al (1997) Human cytomegalovirus infection up-regulates interleukin-8 gene expression and stimulates neutrophil transendothelial migration. Immunology 92: 138–145. 9370936 10.1046/j.1365-2567.1997.00310.x PMC1363993

[4] Hoffmann E, Dittrich-Breiholz O, Holtmann H, Kracht M (2002) Multiple control of interleukin-8 gene expression. J LeukocBiol 72: 847–855. 12429706

[5] Kasahara T, Mukaida N, Yamashita K, Yagisawa H, Akahoshi T, et al (1991) IL-1 and TNF-alpha induction of IL-8 and monocyte chemotactic and activating factor (MCAF) mRNA expression in a human astrocytoma cell line. Immunology 74: 60–67. 1937574 PMC1384672

[6] Aihara M, Tsuchimoto D, Takizawa H, Azuma A, Wakebe H, et al (1997) Mechanisms involved in Helicobacter pylori-induced interleukin-8 production by a gastric cancer cell line, MKN45. Infect Immun 65: 3218–3224. 9234778 10.1128/iai.65.8.3218-3224.1997 PMC175455

[7] Medin CL, Rothman AL (2006) Cell type-specific mechanisms of interleukin-8 induction by dengue virus and differential response to drug treatment. J Infect Dis 193: 1070–1077. 16544247 10.1086/502630

[8] Khabar KS, Al-Zoghaibi F, Al-Ahdal MN, Murayama T, Dhalla M, et al (1997) The alpha chemokine, interleukin 8, inhibits the antiviral action of interferon alpha. J Exp Med 186: 1077–1085. 9314556 10.1084/jem.186.7.1077 PMC2199072

[9] Perkins ND (2007) Integrating cell-signalling pathways with NF-kappaB and IKK function. Nat Rev Mol Cell Biol 8: 49–62. 17183360 10.1038/nrm2083

[10] Miyamoto S (2011) Nuclear initiated NF-κB signaling: NEMO and ATM take center stage. Cell Res 21: 116–130. 21187855 10.1038/cr.2010.179 PMC3193401

[11] Abraham RT (2001) Cell cycle checkpoint signaling through the ATM and ATR kinases. Genes Dev 15: 2177–2196. 11544175 10.1101/gad.914401

[12] Wang SK, Duh CY, Wu CW (2004) Human cytomegalovirus UL76 encodes a novel virion-associated protein that is able to inhibit viral replication. J Virol 78: 9750–9762. 15331708 10.1128/JVI.78.18.9750-9762.2004 PMC515012

[13] Davison AJ, Dargan DJ, Stow ND (2002) Fundamental and accessory systems in herpesviruses. Antiviral Res 56: 1–11. 12323395 10.1016/s0166-3542(02)00107-9

[14] Knizewski L, Kinch L, Grishin NV, Rychlewski L, Ginalski K (2006) Human herpesvirus 1 UL24 gene encodes a potential PD-(D/E)XK endonuclease. J Virol 80: 2575–2577. 16474163 10.1128/JVI.80.5.2575-2577.2006 PMC1395385

[15] Murzin AG, Brenner SE, Hubbard T, Chothia C (1995) SCOP: a structural classification of proteins database for the investigation of sequences and structures. J MolBiol 247: 536–540. 10.1006/jmbi.1995.0159 7723011

[16] Yu D, Silva MC, Shenk T (2003) Functional map of human cytomegalovirus AD169 defined by global mutational analysis. ProcNatlAcadSci U S A 100: 12396–12401. 10.1073/pnas.1635160100 PMC218769 14519856

[17] Isomura H, Stinski MF, Murata T, Nakayama S, Chiba S, et al (2010) The human cytomegalovirus UL76 gene regulates the level of expression of the UL77 gene. PLoS One 5: e11901. 20689582 10.1371/journal.pone.0011901 PMC2912765

[18] Nascimento R, Dias JD, Parkhouse RM (2009) The conserved UL24 family of human alpha, beta and gamma herpesviruses induces cell cycle arrest and inactivation of the cyclinB/cdc2 complex. Arch Virol 154: 1143–1149. 19526192 10.1007/s00705-009-0420-y

[19] Nascimento R, Parkhouse RM (2007) Murine gammaherpesvirus 68 ORF20 induces cell-cycle arrest in G2 by inhibiting the Cdc2-cyclin B complex. J Gen Virol 88: 1446–1453. 17412972 10.1099/vir.0.82589-0

[20] Siew VK, Duh CY, Wang SK (2009) Human cytomegalovirus UL76 induces chromosome aberrations. J Biomed Sci 16: 107. 19930723 10.1186/1423-0127-16-107 PMC2788540

[21] Wu ZH, Shi Y, Tibbetts RS, Miyamoto S (2006) Molecular linkage between the kinase ATM and NF-kappaB signaling in response to genotoxic stimuli. Science 311: 1141–1146. 16497931 10.1126/science.1121513

[22] Murayama T, Ohara Y, Obuchi M, Khabar KS, Higashi H, et al (1997) Human cytomegalovirus induces interleukin-8 production by a human monocytic cell line, THP-1, through acting concurrently on AP-1- and NF-kappaB-binding sites of the interleukin-8 gene. J Virol 71: 5692–5695. 9188651 10.1128/jvi.71.7.5692-5695.1997 PMC191819

[23] Gaspar M, Shenk T (2006) Human cytomegalovirus inhibits a DNA damage response by mislocalizing checkpoint proteins. ProcNatlAcadSci U S A 103: 2821–2826. 10.1073/pnas.0511148103 PMC1413835 16477038

[24] Luo MH, Rosenke K, Czornak K, Fortunato EA (2007) Human cytomegalovirus disrupts both ataxia telangiectasia mutated protein (ATM)- and ATM-Rad3-related kinase-mediated DNA damage responses during lytic infection. J Virol 81: 1934–1950. 17151099 10.1128/JVI.01670-06 PMC1797560

[25] Penfold ME, Dairaghi DJ, Duke GM, Saederup N, Mocarski ES, et al (1999) Cytomegalovirus encodes a potent alpha chemokine. ProcNatlAcadSci U S A 96: 9839–9844. 10.1073/pnas.96.17.9839 PMC22297 10449781

[26] Lüttichau HR (2010) The cytomegalovirus UL146 gene product vCXCL1 targets both CXCR1 and CXCR2 as an agonist. J BiolChem 285: 9137–9146. 10.1074/jbc.M109.002774 PMC2838333 20044480

[27] E X, Pickering MT, Debatis M, Castillo J, Lagadinos A, et al (2011) An E2F1-mediated DNA damage response contributes to the replication of human cytomegalovirus. PLoSPathog 7: e1001342. 10.1371/journal.ppat.1001342 PMC3093362 21589897

[28] Zandi E, Chen Y, Karin M (1998) Direct phosphorylation of IkappaB by IKKalpha and IKKbeta: discrimination between free and NF-kappaB-bound substrate. Science 281: 1360–1363. 9721103 10.1126/science.281.5381.1360

[29] Brockman JA, Scherer DC, McKinsey TA, Hall SM, Qi X, et al (1995) Coupling of a signal response domain in I kappa B alpha to multiple pathways for NF-kappa B activation. Mol Cell Biol 15: 2809–2818. 7739562 10.1128/mcb.15.5.2809 PMC230512

