# Supplementary figures and images for: Human Cytomegalovirus Gene UL76 Induces IL-8 Expression through Activation of the DNA Damage Response
Source: PLoS Pathog. 2013 Sep 12;9(9):e1003609. doi: 10.1371/journal.ppat.1003609 (PMC3771893; doi:10.1371/journal.ppat.1003609)

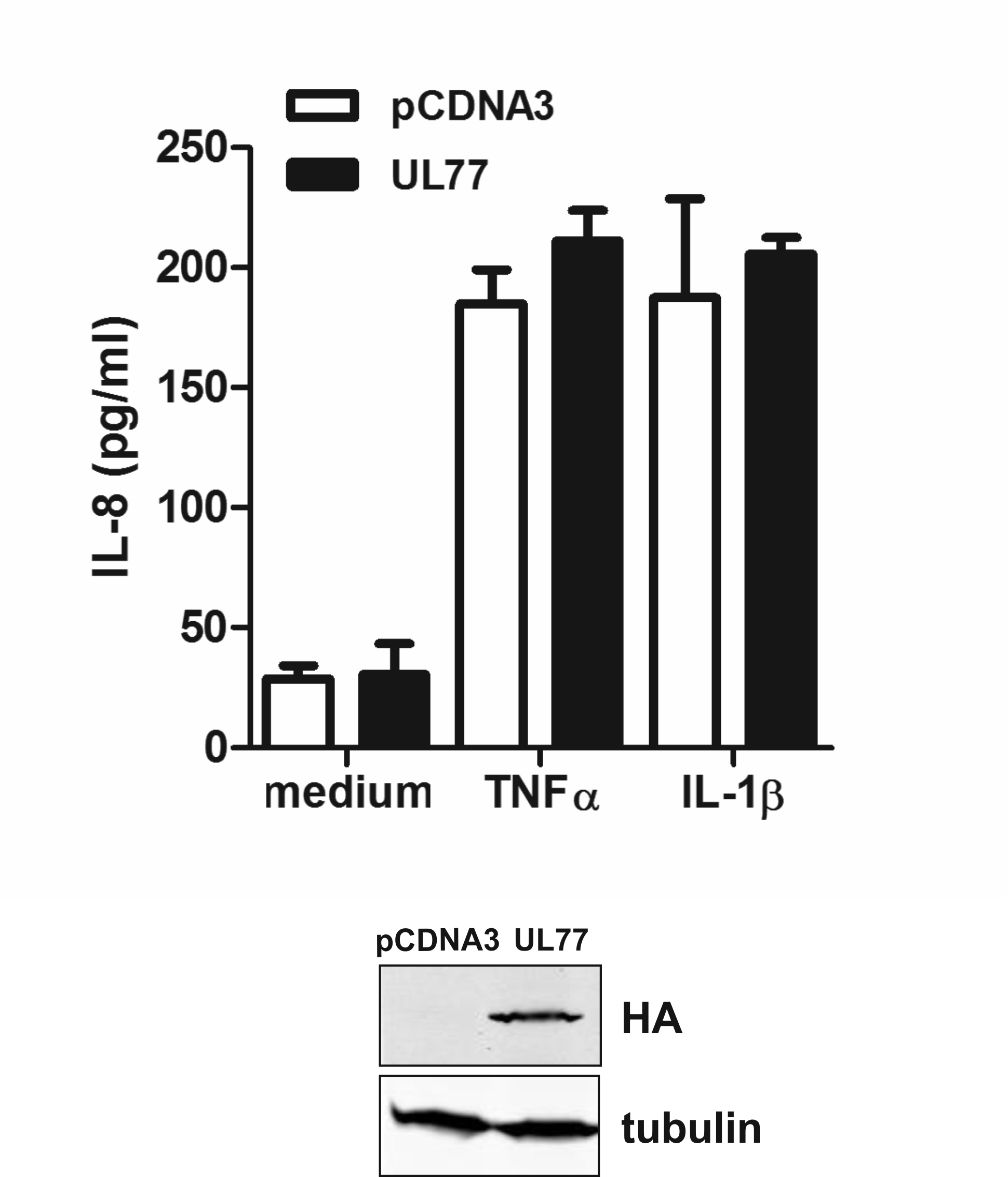

Supplement: Figure S1 — UL77 has no effect in the expression of IL-8. IL-8 concentration in supernatants of 293T cells transfected with control plasmid or pcDNA3HA-UL77 was determined 48 h post-transfection by ELISA, before and after stimulation for 5 h with TNFα (10 ng/ml) or IL-1β (1 ng/ml). Data are expressed as means ± SD of duplicate wells from one of two similar experiments. Expression of HA-tagged UL77 protein was confirmed by western blot (below) using an anti-HA antibody and β-actin detection was used as loading control. (TIF) [file ppat.1003609.s001.tif]

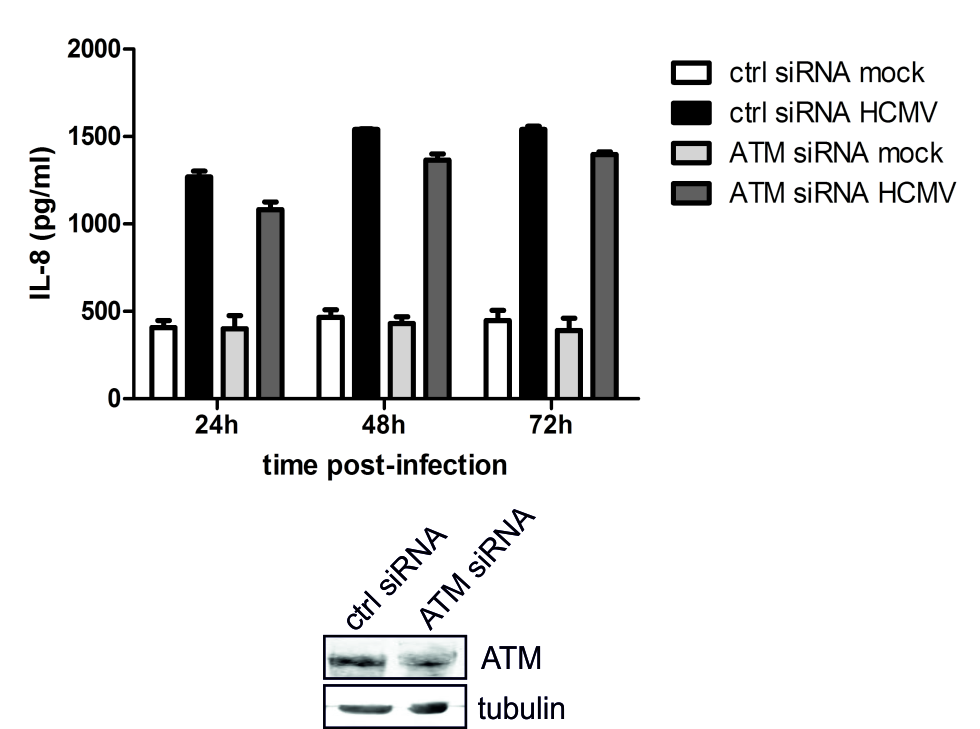

Supplement: Figure S2 — Impact of ATM on the induction of IL-8 by HCMV. A) Primary fibroblasts ATM -/- cells (GM01588) were infected with HCMV wild type (wt) or UL76 mutant virus (TNUL76) at a MOI of 3. Supernatants were collected at 24 h, 48 h and 72 h post-infection and IL-8 concentration was determined by ELISA. Data are expressed as means ± SD of fold induction to mock-infected cells and are representative of three similar experiments. Cells were lysed at 72 h post-infection and viral infection was confirmed by western blot (below) using an anti-UL44 antibody for the CMV viral protein and detection of β-actin as loading control. B) HFF cells were transfected with ON-TARGETplus Human ATM siRNA or control siRNA (ctrl) (100 µM) 24 h prior to infection with HCMV (MOI 3). Supernatants were collected at 24 h, 48 h and 72 h post-infection and IL-8 concentration was determined by ELISA. Data are expressed as means ± SD and are representative of two similar experiments. Levels of ATM protein in cells transfected with ATM siRNA or control siRNA were determined by western blot using an anti-ATM antibody and detection of tubulin as loading control. (TIF) [file ppat.1003609.s002.tif]
